# Malaria, medicines and miles: A novel approach to measuring access to treatment from a household perspective

**DOI:** 10.1016/j.ssmph.2019.100376

**Published:** 2019-03-06

**Authors:** Benjamin Palafox, Catherine Goodman, Kara Hanson

**Affiliations:** Department of Global Health & Development, London School of Hygiene & Tropical Medicine, 15-17 Tavistock Place, London WC1H 9SH, United Kingdom

**Keywords:** ACT, artemisinin combination therapy, AETD, adult equivalent treatment dose, AMFm, Affordable Medicines Facility–malaria, AMT, artemisinin monotherapy, CI, 95% confidence interval, DHS, Demographic and Health Survey, IQR, interquartile range, nAT, non-artemisinin therapy, PPMV, proprietary patented medicine vendor, PSU, primary sampling unit, RDT, rapid diagnostic test (for malaria), USD, United States dollar, WHO, World Health Organization, Antimalarials, Malaria, Access to healthcare, Private sector, Public sector, Health Equity, Population metrics

## Abstract

Nearly a decade after the adoption of confirmed diagnosis and artemisinin combination therapy (ACT) for the treatment of uncomplicated falciparum malaria, a large treatment gap persists. We describe a novel approach of combining data from households and the universe of treatment sources in their vicinities to produce nationally representative indicators of physical and financial access to malaria care from the household’s perspective in Benin, Nigeria, Uganda and Zambia. We compare differences in access across urban and rural areas, countries, and over time.

In 2009, more urban households had a provider stocking ACT within 5 km than rural households. By 2012, this physical ACT access gap had largely been closed in Uganda, and progress had been made in Benin and Nigeria; but the gap persisted in Zambia. The private sector helped to fill this gap in rural areas. Improvements in Nigeria and Uganda were driven largely by increased ACT availability in licensed drug stores, and in Benin by increased availability in unregulated open-air market stalls. Free or subsidised ACT from public and non-profit facilities continued to be available to many households by 2012, but much less so in rural areas. Where private sector expansion increased physical access to ACT, these additional options were on average more expensive. Also by 2012, the majority of urban households in all four countries had access to a provider nearby offering malaria diagnostic services; however, this access remained low for rural households in Benin, Nigeria and Zambia.

The methods developed in this study could improve how access to healthcare is measured in low- and middle-income country settings, particularly where private for-profit providers are an important source of care, and for conditions that may be treated by informal providers. The method could also lead to better explanations of the performance of complex interventions aiming to improve healthcare access.

## Highlights

• Common measures of healthcare access are often limited to demand- or supply-side descriptions.
• We present a novel approach to produce indicators of access to care from the household perspective.
• The developed methods could improve how access to healthcare is measured.
• The methods could also help to understand how complex interventions improve healthcare access.

## 1 Introduction

In 2016, an estimated 216 million cases of malaria worldwide led to more than 445,000 deaths, mostly among children in sub-Saharan Africa (WHO Global Malaria Programme, 2017). Effective management for uncomplicated cases of *Plasmodium falciparum* malaria, the species causing the majority of fatal infections, requires confirmed diagnosis and, if positive, treatment with artemisinin combination therapy (ACT), ideally prescribed and dispensed from a qualified provider. However, access to appropriate diagnosis and treatment is still inadequate, resulting in a large treatment gap where many cases are managed sub-optimally or even go untreated.

To illustrate, although nearly a decade has passed since the World Health Organization (WHO) updated its guidelines for treating uncomplicated falciparum malaria to recommend confirmed diagnosis and ACT, it is estimated that among febrile children in sub-Saharan Africa for whom care was sought, only 30% received a diagnostic test either by microscopy or rapid diagnostic test (RDT) in 2014-16 (WHO Global Malaria Programme, 2017). Moreover, many of those with malaria do not receive ACT. For example, studies in Tanzania found that among cases positive for malaria by reference blood slide, just over half (50.2%) received ACT in government facilities and less than a third of those who sought care from private drug stores (Briggs et al., 2014, Bruxvoort et al., 2013). Many more were not brought for care or received older non-artemisinin therapies (nATs), such as sulphadoxine-pyrimethamine and chloroquine. Widespread parasite resistance has rendered nATs less effective in many endemic regions of sub-Saharan Africa (Okell et al., 2017, Takala-Harrison and Laufer, 2015).

Poor coverage of effective treatment persists despite considerable investment by Ministries of Health and their partners in a range of strategies to address the issue. These interventions typically aim to reduce critical access barriers in various ways. For example, efforts have been made to increase ACT and RDT availability at government facilities, dispense them free of charge from public sector outlets, train community health workers and private retailers (i.e. pharmacies and drug stores) to conduct diagnostic testing and dispense appropriate treatment, curtail the retailing of non-ACT, and lower private sector ACT prices through subsidies (Global Fund, 2016, Kabaghe et al., 2016, Rao et al., 2013, Visser et al., 2017). The most notable of these subsidy programmes was the Affordable Medicines Facility–malaria (AMFm), which was piloted in several endemic countries, including Nigeria and Uganda, from 2010–12 (Tougher et al., 2012). The AMFm was a multi-national subsidy programme for ACTs implemented at a national scale in 7 African countries, funded by the Global Fund to Fight HIV, TB and Malaria. It aimed to increase the appropriate use of quality-assured ACTs and decrease the use of other antimalarials, through a combination of ACT subsidies and supporting interventions such as recommended retail prices and communications campaigns. However, despite all these efforts, the size of the treatment gap indicates that much more still needs to be done to improve malaria case management.

Within the international public health community, it is now widely acknowledged that access to health care is a multi-dimensional concept based on the interaction or ‘degree of fit’ or ‘alignment’ between health care systems and individual, household, and community needs, which may either empower or hinder an individual’s use of appropriate health care (Lévesque et al., 2013, McIntyre et al., 2009). One such definition developed by McIntyre and colleagues categorises the many factors that determine access into three dimensions: availability or physical access, affordability or financial access, and acceptability or cultural access (McIntyre et al., 2009). In addition, it has been argued that efforts to understand health care use must account for the broader range of treatment options that an individual might engage with, beyond official medical sources (MacKian, Bedri, & Lovel, 2004). This is of particular relevance for malaria treatment, which is known to involve a diverse array of providers ranging from public, private and faith-based health care facilities, to retail pharmacies, drug stores, and general retailers (Littrell et al., 2011).

Efforts to define health care access have tended to focus on its conceptualisation, without much attention to the application of the concept or its measurement (McIntyre et al., 2009). Indeed, this is evident in many of the common indicators used to measure access. For example, access indicators derived from household data such as Demographic and Health Surveys (DHS), Multiple Indicator Cluster Surveys and Malaria Indicator Surveys (ICF International, 2015, Roll Back Malaria Partnership, 2013, UNICEF, 2015), may provide information on distance or travel time to the chosen health care provider, the type of treatment obtained and its price. However, these indicators do not give information on the broader range of provider options available to that individual or to individuals not seeking treatment, or on the range and price of the alternative treatments that providers offer. In particular, these data do not reveal whether a household has at least one provider in their area with the required health products and services. In the case of malaria, such information is critical to understand the choice not to seek care or to obtain sub-optimal treatment.

On the other hand, health care facility or provider surveys and administrative datasets may be able to provide comprehensive descriptions of the supply side. For example, the Service Provision Assessment surveys offered by the DHS collect data on the specific health services offered by facility type and whether these facilities have the necessary infrastructure, resources and support systems available (ICF International, 2015). However, such average data on provider readiness cannot reveal what a given household’s access to these services actually is, especially as the better performing facilities may be clustered geographically, and such assessments rarely include all provider types. Such indicators also cannot also account for how well accessible health care products and services align with actual need.

Therefore, measures of access that combine both household and provider data to characterise all the treatment options available to an index household could substantially improve our understanding of health care access. This paper describes a novel method to develop such measures by combining supply- and demand-side survey data from the ACTwatch project to produce nationally representative indicators of access to care for malaria. We demonstrate the utility of this approach by estimating a select range of physical and financial access indicators that characterise the malaria treatment options available to households with a febrile child in Benin, Nigeria, Uganda and Zambia, and use these to describe how access has changed over time.

## 2 Methods

### 2.1 Data and source

The ACTwatch project was designed to generate nationally representative information on antimalarial markets through linked cross-sectional surveys of households, treatment sources and private sector distribution chains in selected endemic countries (Shewchuk et al., 2011). Participating countries were chosen to represent a diverse range of contexts considering variation in malaria burden, the nature of pharmaceutical regulation (e.g. high vs. low regulatory capacity; francophone vs. anglophone settings), public sector coverage, and domestic antimalarial manufacturing capacity.

This study uses household and treatment source data from two survey rounds conducted in Benin, Nigeria, Uganda and Zambia. We selected two countries which had received the AMFm antimalarial subsidy (Nigeria and Uganda) and two which had not (Benin and Zambia). The first round (baseline) was conducted in 2009-10 (pre-AMFm), and the second round (endline) in 2011-12 (during AMFm). In each country and during each round, household and treatment source surveys were conducted contemporaneously using a common multi-staged clustered sampling design. Briefly, national samples of households and treatment sources were drawn from the same primary sampling units (PSUs), within which every treatment source that had recently stocked an antimalarial (i.e. within the preceding three months) was eligible for inclusion. Treatment sources were identified using a census approach and included public and not-for-profit health facilities, private (for-profit) health facilities, retail pharmacies, drug stores (apart from in Benin; also known as Patent Proprietary Medicine Vendors, or PPMVs, in Nigeria), and general retailers, such as grocery stores, kiosks and market stalls (O’Connell et al., 2011). As public health facilities and pharmacies are important, but relatively uncommon sources of antimalarials, these provider types were over-sampled by including such providers in the larger administrative area from which a given PSU was selected. For example, if the PSU was defined as the sub-district, all public health facilities and pharmacies in the whole district within which the sub-district was located were sampled.

Households containing a recently febrile child were randomly selected from three secondary sampling units drawn within each PSU surveyed. The household surveys collected demand-side information on the treatment choices made for febrile children, and on personal and household characteristics, while the treatment source surveys collected supply-side information on the availability and price of all antimalarials and all malaria diagnostics, and other provider characteristics related to staffing, storage conditions, knowledge, etc. Geographic coordinates were also collected from all surveyed households and treatment sources. Table 1 provides details of the household and treatment source samples in each country (with breakdown by rural/urban strata in Appendix Table A, Table B); sampling and survey procedures are described elsewhere (Littrell et al., 2011, O’Connell et al., 2011, Shewchuk et al., 2011).

**Table 1:** Characteristics of the household and treatment source samples by survey round and country.

	**Benin**		**Nigeria**		**Uganda**		**Zambia**	
**Survey round**	1	2	1	2	1	2	1	2
**Survey Date**	Apr-Jul 2009	Apr-Jul 2011	Aug-Nov 2009	Oct 2011-Jul 2012	Mar-Apr 2009	Nov 2011-May 2012	Apr-Jul 2009	Mar-Sep 2011
**Number of strata**	1	1	6	2	2	2	2	2
Total clusters	19	19	110	76	38	44	38	38
**Total households screened**	2312	3275	4616	7863	2063	5067	2218	*
Number of households with a febrile child under 5 years	874	904	2802	1310	1523	1826	1693	1205
Number of households with multiple febrile cases	63	117	469	216	266	403	188	132
**Total febrile children under 5 years surveyed**	927	1015	3274	1551	1753	2274	1886	1341
Number of cases receiving an antimalarial	378	533	1060	565	899	1177	709	555
Number of cases for whom no initial treatment was sought	161	31	129	26	78	61	172	109
Number of cases initially treated at home	409	556	1046	732	615	1376	455	317
Number of cases seeking care from multiple external sources	36	102	302	162	143	309	119	188
**Number of outlets screened**	1670	2738	5456	7939	5267	16 207	3378	5436
Number of ineligible outlets	609	1348	3246	6372	3989	12 922	2917	4565
Number of outlets surveyed	1061	1390	2196	1562	1278	3227	461	861
Number of outlets stocking ACT	299	588	1168	947	615	2697	240	474
Number of outlets stocking nAT	828	1119	2042	1433	1159	2920	424	742
Number of outlets stocking oral AMT	46	5	1085	551	167	1	26	8
								
**Number of antimalarial tablets inventoried**								
Number of ACTs	2023	4009	4614	3254	1162	8770	708	1446
Number of nATs	1730	2220	6565	4289	2275	5083	727	1164
Number of oral AMTs	59	5	1623	697	237	1	20	8

* Households not meeting eligibility criteria were not included in the household survey dataset

### 2.2 Producing the access dataset and indicators

In each country, we merged household and treatment source data for each survey round to create the access datasets. For each surveyed household with a recently febrile child, a treatment choice set was defined by forming pairwise links between each household and all treatment sources surveyed that lay within a 5 km radius, reflecting likely willingness to travel for antimalarial providers, as noted in the literature (Noor et al., 2006, Noor et al., 2003, Toda et al., 2012). Straight-line distances between households and paired treatment sources were calculated using the *geodist* package in Stata 13 (StataCorp, 2013), and sources located more than 5 km away were removed from that household’s treatment choice set. A 5 km radius was chosen to approximate a one-hour walking distance, which has been used previously to denote reasonable geographic access in developing country contexts (Skiles et al., 2013, Smith et al., 1999, Tanser, 2006). Defining treatment choice sets in this way potentially introduces a measurement bias when surveyed households are located close to the border of the PSU. This would result in underestimation of access because treatment options close to the household, but outside the PSU border, would be excluded from the choice set. Such bias is less for public health facilities and pharmacies as these were oversampled from a larger area surrounding the PSU; however, statistical comparisons between these and other provider types are not appropriate given the difference in sampling approaches.

Using the merged household and treatment source data we characterise health care access from the perspective of households. In this paper, we present the following nationally representative indicators by urban and rural location, and by survey round, for an access area defined by a 5 km radius around households: % households with access to any treatment source stocking any antimalarial; % households with access to any treatment source stocking ACT; % households with access to ACT by type of treatment source; % households with access to any treatment source offering malaria diagnostic services; % households with access to any treatment source staffed by a qualified health professional (i.e. medical doctor, nurse, midwife, pharmacist); % households with access to any treatment source with ACT, offering diagnostic services and staffed by a qualified health professional; median number of treatment sources stocking ACT; and median price for ACT, nAT and oral artemisinin monotherapy (AMT) tablets.

### 2.3 Statistical analysis

Indicator estimates were adjusted using inverse-probability sampling weights to account for differences in the household probability of being selected in those countries where samples were stratified. Standard error estimation accounted for clustering within primary and secondary sampling units. Thus, estimates are conservative and reduce the likelihood of incorrectly rejecting the null hypothesis.

Percentage-based estimates are presented with 95% confidence intervals (CI), and differences in percentages over time are tested against the null hypothesis of no change. Prices for ACT tablets, the most common dosage form used for treatment, are presented in adult equivalent treatment doses (AETDs), a standardised unit that allows for comparison of products with different treatment regimens. Prices were adjusted to a 2010 base using the World Bank annual consumer price index values, and converted to US dollars (USD) using the average weekly exchange rate in 2010 (O’Connell et al., 2011, Tougher et al., 2012). Price-based indicators are estimated as medians with interquartile range (IQR), and differences over time are examined using the Wilcoxon rank-sum test. Analyses were conducted in Stata 13 and R version 3.0.2 (StataCorp, 2013, The R Foundation for Statistical Computing, 2013).

## 3 Results

### 3.1 Physical access to ACT and other antimalarials

**Fig. 1:**
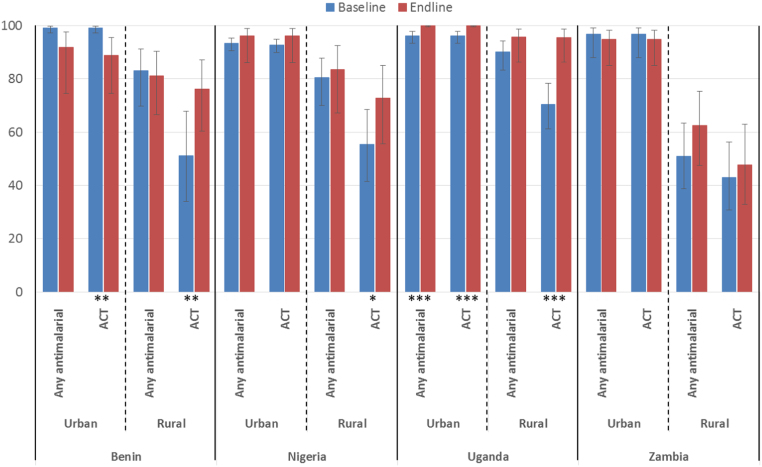
Percentage and 95% confidence interval of households with access to ACT and any antimalarial within 5 km by urban-rural location and over time, with 95% confidence interval (For differences in percentages over time: * p≤0.1, ** p≤0.05, *** p≤0.001).

Differences in physical access to ACTs and other antimalarials were observed across urban and rural areas and over time. In urban areas, ACTs were accessible to the majority of households. In all four countries both at baseline and endline, more than 85% of urban households had access to at least one source of ACTs within a 5 km radius (Fig. 1). Physical access to ACTs in rural areas was lower than in urban areas; however, in Benin, Nigeria and Uganda, household access to ACTs in rural areas improved over time. For example, the percentage of rural households in Benin with access to at least one source of ACTs within 5 km increased from 51% to 76% (p-value: 0.023) between surveys. In Uganda, the urban-rural ACT access gap had largely been eliminated by 2012 where over 95% of households in both urban and rural areas had access to at least one source of ACT within 5 km, whereas in Zambia, household access did not improve in rural areas between baseline and endline. In addition, no notable changes in physical access to any antimalarial were observed over time in any of the study countries. As such, the significant improvements in ACT access noted in rural areas of Benin, Nigeria and Uganda may indicate that over time existing sources previously stocking only non-ACTs have begun to stock ACT as well.

Our analysis was based on repeated cross-sectional surveys rather than panel data (i.e. different PSUs were selected in each round). While each round was designed to be nationally representative, it is possible that the selection of outliers in terms of PSU characteristics in one round could have influenced estimates of change over time. Inspection of sample characteristics disaggregated by urban/rural strata (Appendix Table A, Table B) shows that there were no substantial differences in average outlet numbers per PSU in all cases except Uganda, where at endline the urban PSUs selected had a higher average number of antimalarial outlets than those selected at baseline (Appendix Table A, Table B). However, this does not affect the patterns described above as the only notable improvements in access in Uganda were seen in rural areas.

### 3.2 Physical access to ACT by treatment source

**Fig. 2:**
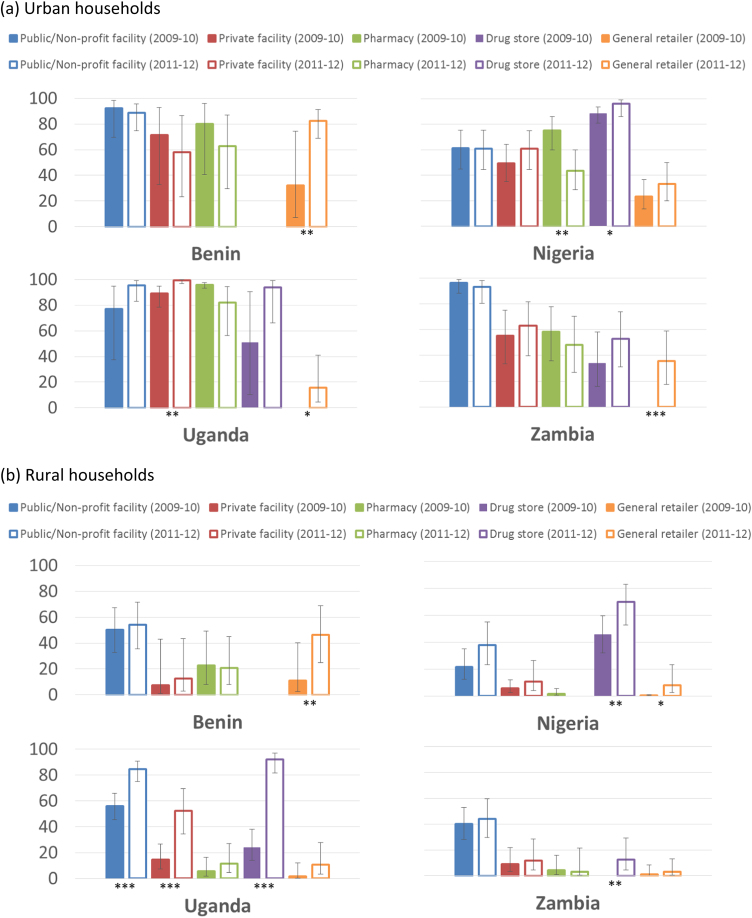
Percentage and 95% confidence interval of (a) urban and (b) rural households with access to ACT within 5 km by treatment source and over time, with 95% confidence interval (For differences in percentages over time within sources: * p≤0.1, ** p≤0.05, *** p≤0.001. Notes: Drug stores were not surveyed in Benin as they are not a common provider type; comparisons across types of treatment source may not be appropriate as public facilities and pharmacies were oversampled in ACTwatch surveys).

Examining changes in the composition of treatment sources within the vicinity of households provides further information on factors driving the improvements in physical access to ACTs described above (Fig. 2). During both survey rounds, many urban households had access to a variety of ACT sources within a 5 km radius, including public and not-for-profit facilities, private for-profit facilities and retail pharmacies (Fig. 2a). Nearly all urban households in Nigeria had access to ACTs via drug stores, which were also common options for households in Uganda and Zambia. In contrast, households in rural areas had fewer options to access ACTs (Fig. 2b). Public and not-for-profit health facilities provided access to ACTs to over half of rural households only in Benin and Uganda, but were still the most common option in rural Zambia. Drug stores were the dominant source of ACT accessible to rural households in Nigeria and for a considerable proportion of households in Uganda.

Over time, there is some evidence that the proportion of urban households with access to ACTs increased through private for-profit facilities in Uganda (77% to 96%, p-value: 0.011); drug stores in Nigeria (89% to 96%, p-value: 0.058); and unlicensed general retailers in Benin (32% to 83%, p-value: 0.014), Uganda (0% to 16%, p-value: 0.064) and Zambia (0% to 36%, p-value: 0.001). In the four countries, there were no significant increases in household access to ACTs through public or not-for-profit providers observed in urban areas; however, in rural areas of Uganda, the proportion of households with access to ACT through these providers increased from 56% to 85% (p-value: <0.001). There is also evidence that rural household access improved through private health facilities in Uganda (15% to 52%, p-value: <0.001); drug stores in Nigeria (46% to 70%, p-value: 0.020), Uganda (24% to 92%, p-value: <0.001) and Zambia (0% to 12%, p-value: 0.029); and unlicensed general retailers in Benin (11% to 46%, p-value: 0.015) and Nigeria (1% to 8%, p-value: 0.094).

In Table 2, we present figures for the median number of treatment sources stocking ACT within 5 km of households over time. In Uganda, the number of ACT providers that a typical household could choose from increased eightfold between 2009-10 and 2011-12, from a median of 10 to 80 providers for urban households, and from a median of 1 to 8 providers for rural households. More modest increases were observed for rural households in Benin and Nigeria, where the median rose from 1 in 2009-10 to 2 by 2011-12. There were no notable changes in rural Zambia. These households had access to a median of 0 treatment sources stocking ACT within 5 km in both survey rounds.

**Table 2:** Median number of treatment sources stocking ACTs within 5 km of urban and rural households over time, with interquartile range (IQR).

		**Urban**			**Rural**		
		**Median**	**IQR**	**p-value**	**Median**	**IQR**	**p-value**
**Benin**	N	Baseline=269, Endline=241			Baseline=595, Endline=659		
	Baseline	5	(3–28)	0.211	1	(0–3)	0.086
	Endline	20	(9–55)		2	(1–4)	
			
**Nigeria**	N	Baseline=995, Endline=726			Baseline=1739, Endline=580		
	Baseline	10	(6–22)	0.439	1	(0–3)	0.010
	Endline	13	(7–29)		2	(0–7)	
			
**Uganda**	N	Baseline=154, Endline=908			Baseline=1308, Endline=903		
	Baseline	10	(9–13)	<0.001	1	(0–3)	<0.001
	Endline	80	(41–154)		8	(5–13)	
			
**Zambia**	N	Baseline=848, Endline=471			Baseline=841, Endline=600		
	Baseline	11	(4–17)	0.334	0	(0–1)	0.596
	Endline	7	(2–16)		0	(0–1)	

### 3.3 Physical access to malaria diagnostic services and qualified health professionals

**Fig. 3:**
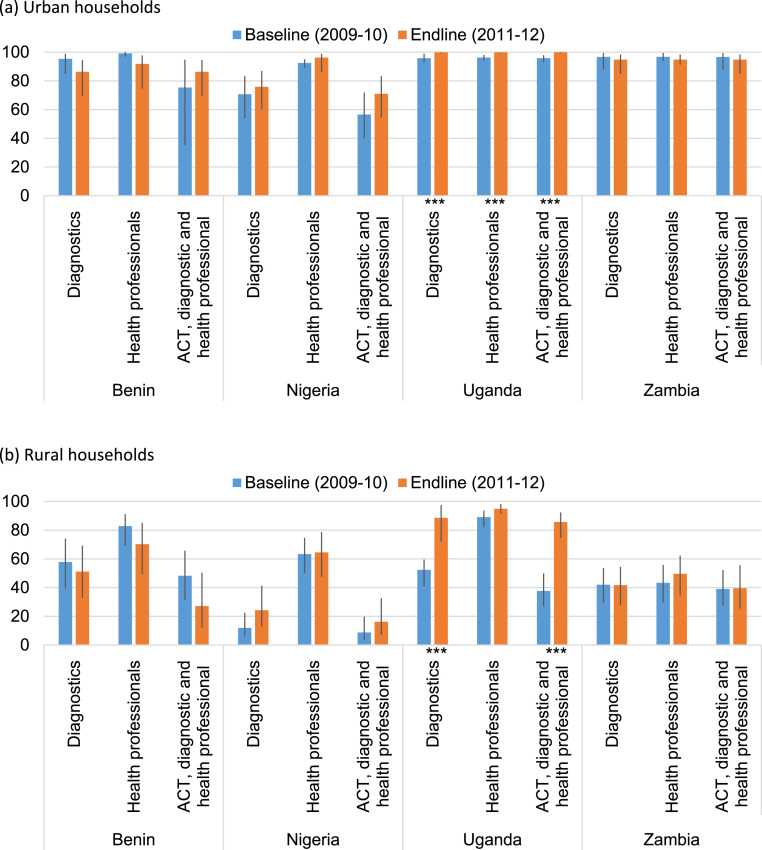
Percentage and 95% confidence interval of (a) urban and (b) rural households with access to antimalarial providers with malaria diagnostics; health professionals; and ACT, diagnostics and health professionals within 5 km (For differences in percentages over time within sources: * p≤0.1, ** p≤0.05, *** p≤0.001. Note: Drug stores were not surveyed in Benin as they are not a common provider type).

Urban households had ready access to health professionals and malaria diagnostic services (Fig. 3a). More than 90% of these households in all four study countries had at least one antimalarial source staffed by a qualified health professional within 5 km, and similar proportions of urban households also had at least one antimalarial source that offered malaria diagnostic services within 5 km in all countries except Nigeria. In rural areas, households in Uganda had a comparable level of access to health professionals as in urban areas, but access to health professionals was lower in rural areas in the remaining countries (Fig. 3b). Household access to diagnostic services in rural areas was poor at just over 50% of households in Benin and over 40% in Zambia. Rural access to diagnostics in Nigeria increased from 12% to 24% of households between surveys (though this change was not statistically significant); and increased from 52% to 89% of rural households in Uganda (p-value: <0.001).

Fig. 3 also illustrates changes in household access within 5 km to any treatment sources stocking both ACT and malaria diagnostics, and staffed by a qualified health professional - the three core elements of malaria case management. More than half of urban households in all four countries had access to at least one of these providers in 2009-10 and 2011-12, and more than 90% of urban households in Uganda and Zambia (Fig. 3a). This access was much lower for rural households (Fig. 3b). Less than a quarter of rural households in Benin and Nigeria, and approximately 40% in rural Zambia had access to any treatment source stocking both ACT and malaria diagnostics, and staffed by a qualified health professional in 2009-10 and 2011-12. Over time improved access to these providers was observed only in rural Uganda, where the proportion of households with such access increased from 38% in 2009-10 to 86% by 2011-12 (p<0.001).

### 3.4 Price of antimalarials accessible to households

**Fig. 4:**
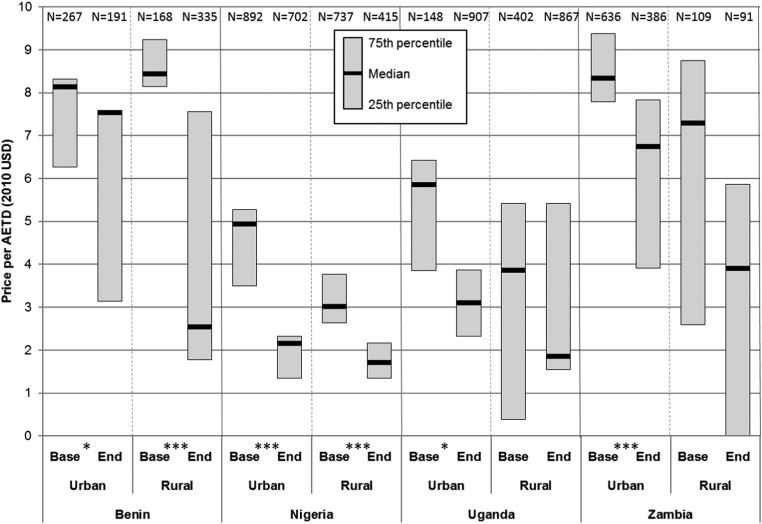
Median price and interquartile range of private sector ACT tablets (2010 USD) per adult equivalent treatment dose within 5 km of urban and rural households over time (For differences in median over time: * p≤0.1, ** p≤0.05, *** p≤0.001).

Because public and not-for-profit health facilities typically dispense ACT free of charge or at heavily subsidised prices (as in Benin), low-cost ACT was accessible to a large majority of urban households in the study countries with access via these treatment sources (Fig. 2a). In contrast, fewer rural households had ready access to affordable ACTs via public and not-for-profit health facilities (Fig. 2b), which could result in delayed treatment or seeking care from more expensive private for-profit providers. Fig. 4 illustrates the median price of ACT (per AETD, 2010 USD) available from private for-profit providers only among those households with access to them by country, urban-rural location and over time.

At baseline, the median price of private sector ACT tablets accessible to urban households ranged from 4.94 USD per AETD in Nigeria to 8.33 USD in Zambia, and for rural households from 3.01 USD in Nigeria to 8.44 USD in Benin. In all countries except Benin, prices for ACT tended to be higher in urban than in rural areas.

Over time, the median prices of ACT accessible to households decreased in all areas of the study countries. However, these changes were statistically significant for urban and rural households in Nigeria (rural: from 4.94 to 2.16 USD, p-value: <0.001; urban: from 3.01 to 1.71 USD, p-value: <0.001), rural households in Benin (from 8.44 to 2.55 USD, p-value: <0.001), and urban households in Zambia (from 8.33 to 6.75 USD, p-value: 0.001). No significant changes were observed in Uganda. Median prices for private sector AMT and nAT, and addition details for ACT tablets are presented in Appendix Table C.

## 4 Discussion

### 4.1 Summary of findings and policy implications

By combining data from households with information on the complete range of treatment sources in their vicinities, we have produced a variety of nationally representative indicators that describe malaria treatment access from the household perspective, and how this has changed over time in dynamic, pluralistic health care markets. We characterise two out of the three dimensions of access (i.e. physical and financial), but do not address the acceptability dimension.

Our findings show that by 2011-12, although the urban-rural gap in physical ACT access persisted in Zambia, progress had been made in Benin and Nigeria, and this gap had largely closed in Uganda. The results also demonstrate that the private sector helped reduce this gap in underserved areas. This was seen in Nigeria and Uganda where increased household access was driven largely by increased ACT availability in licensed drug stores, which was in large part due to these countries’ participation in the AMFm ACT subsidy programme that explicitly aimed to increase ACT availability and affordability among private retailers (Tougher et al., 2012). Although Benin did not participate in AMFm previous research has shown that Benin’s private retail sector is heavily dependent on Nigeria for antimalarial supplies (Palafox et al., 2014), so that the impact of AMFm was indirectly felt in Benin as well. After the official end of AMFm, ACT subsidies were maintained in Nigeria and Uganda under the Private Sector Co-payment Mechanism, which sustained the improvements in access achieved during the AMFm period (ACTwatch Group, Tougher, Hanson, & Goodman, 2017).

However, increasing physical access through private for-profit providers carries potential consequences for the affordability of care. Although we do not measure ability to pay, our data on price are an important component of financial access. To illustrate, access to ACT for rural households in Uganda in 2009-10 was predominantly via public and not-for-profit health facilities where treatment should be dispensed free of charge. The increases in physical access seen by 2011-12 were driven largely through more private for-profit health facilities and drug stores stocking ACT. Although free treatment from public and not-for-profit facilities was still available to rural households in Uganda in 2011-12, individuals may choose to purchase more expensive ACT from private providers because they may be more convenient in terms of proximity, waiting times and opening hours. However, the observed decreases in private sector ACT prices demonstrate the role that interventions, like the AMFm ACT subsidy programme, continue to play in ensuring wide access to more affordable ACTs (ACTwatch Group, Tougher, et al., 2017). Nonetheless, improving physical access to affordable care in public facilities must remain as a core objective of equitable health system strengthening in these settings as even subsidised ACT will still be out of reach for many.

Results from Benin and rural Zambia also illustrate some less desirable effects of broader private sector involvement, where rising ACT availability among general retailers contributed to the observed increase in household access. In Benin, previous studies have shown that unlicensed and unregulated open-air market stalls selling antimalarials dominate this class of provider (O’Connell et al., 2011), among whom it would be difficult to ensure the quality of medicines and case management. On the other hand, it could be argued that it is unrealistic to expect most people to seek care through Benin’s mainly urban private facilities and pharmacies, and therefore, steps should be taken to increase the quality of alternative treatment sources. Such measures could include introducing a category of regulated medicine dispenser akin to drug stores in other countries, support for market stall vendors who wish to upgrade, and training on malaria case management for drug store operators (Goodman et al., 2007, Rao et al., 2013).

While our findings show that the ACT access gap is closing, they also illustrate that, apart from in Uganda, much more could be done to improve rural access to the other core elements of appropriate malaria case management - diagnostics and qualified health professionals. By 2011-12, among rural households in Benin, Nigeria and Zambia, less than 70% had access to a treatment source within 5 km staffed by a qualified health professional, and less than half had access to malaria diagnostics. Access to an outlet within 5 km with all three core elements of malaria case management in rural areas was 40% of households in Zambia, 27% in Benin and only 16% in Nigeria. Public and private health facilities continue to be the dominant providers of diagnostic services in endemic countries (ACTwatch Group, Hanson, & Goodman, 2017). Given the positive impact that engagement with private providers has had on improving access to ACT, careful consideration needs to be given to the role private retailers should play in ensuring that malaria diagnostics, and rapid diagnostic tests in particular, cover the last mile (Visser et al., 2017).

### 4.2 Methodological insights into measuring health care access

We believe that the methods presented here produce indicators of access that improve upon those previously used, and that they have many useful applications. First, we have demonstrated how examining treatment availability and prices charged from the perspective of households provides more intuitive and meaningful descriptions of access. As described in the introduction, typical access indicators are often limited to demand- or supply-side descriptions, typically averaged over broad strata (e.g. urban/rural), while our indicators provide direct measures of the services obtainable within a reasonable distance from households, thus providing evidence on ‘last mile’ coverage (Lévesque et al., 2013, McIntyre et al., 2009).

A further advantage of this approach is that access to health services can be *aligned with actual need*. To do this for our malaria case study, we use information from the ACTwatch household survey on reported fever for a child under the age of 5 years, which other open access resources, such as maps of populations or structures, do not provide. While reported fever in the study countries was widely distributed, this aspect of the approach could be more important for other conditions that are more geographically clustered, such as HIV and tuberculosis.

When such fine-grained descriptions of access are examined over time, these indicators also provide much clearer understanding of whether household access has improved, and the changes driving those improvements. When considered alongside the acceptability dimension of access, this can indicate how such changes may impact treatment utilisation and ultimately, health outcomes. In applying these methods to malaria treatment, we have probed such access changes in Benin, Nigeria and Uganda, and the lack thereof in Zambia. These indicators could then be used as explanatory variables in analysing changes in treatment seeking behaviour. Given this explanatory potential, such indicators could be used to better evaluate the impact of interventions designed to improve access to care.

These particular strengths of our method rely not only on the ability to combine contemporaneous demand- and supply-side data from the same locations, but also on the comprehensiveness of the supply-side data. A number of previous analyses have linked Service Provision Assessment data with DHS household data in novel and interesting ways (Akin et al., 1998, Skiles et al., 2013). However, Service Provision Assessments focus on public and private facilities only and, therefore, do not provide information on the availability of other treatment options, particularly in the retail sector which is such an important source of malaria treatment. By contrast, the ACTwatch treatment source surveys provide a complete picture of the treatment landscape.

However, conducting simultaneous household and treatment source surveys is logistically challenging and costly. This is particularly the case when including all treatment sources as the presence of less qualified providers and retailers is generally not well-documented, meaning that a detailed census must be conducted. Since the end of the ACTwatch project, such total market surveys of malaria treatment sources are not being conducted, so alternative sources of data would need to be found to produce comprehensive assessments of access to malaria treatment. One option could be to expand Service Provision Assessments to include a census of all provider types in PSUs and collect a broader range of supply-side data in countries where DHS household surveys include the malaria module. The methods in this paper could also be applied using data from ACTwatch’s sister project FPwatch, which involves surveys of all family planning providers alongside household surveys (PSI, 2018). Conversely, for cases where concurrent, co-located household data are not available, open source maps of populations or structures could be used to locate households and then be merged with supply-side data from sources such as those described above. However, since these open access resource typically do not include information on actual household need for care, this approach would be better suited to measuring access to care for conditions that are more or less evenly distributed within a population.

Our approach of using a 5 km radius around households to define their treatment choice sets represents a fairly simplistic use of geospatial data, and we recognise that more sophisticated methods, such as those that estimate road distance and travel time, could be used to assess physical access (Al-Taiar et al., 2010, Islam and Aktar, 2011). As described in methods, defining treatment choice sets in this way also risks underestimating access for households located close to the borders of sampled PSUs, although the effect of this bias could be minimised by reducing the radius length used. Other important biases to consider are those typical when using self-reported data. For example, social desirability bias may have led to under-reporting of antimalarial prices and the availability of undesirable treatments by providers (i.e. oral AMT). While a methodological strength of the approach is the full census of treatment sources in PSUs, some providers with the potential to sell antimalarials may have been missed, leading to underestimates of treatment availability.

## 5 Conclusion

In this paper, we have described an approach to operationalise the physical and financial dimensions of access to health care from the household perspective. Applying these methods to examine access to malaria treatment in four endemic countries, we have also illustrated how this novel approach provides a more useful understanding of access to care in pluralist and varied health care markets. This approach also facilitates an understanding of the drivers of changes in access over time and could lead to better explanations of the performance of complex interventions aiming to improve healthcare access.

## Figures and Tables

**Table A:** Characteristics of the urban household and treatment source samples by survey round and country.

	**Benin**		**Nigeria**		**Uganda**		**Zambia**	
**Survey round**	1	2	1	2	1	2	1	2
**Number of strata**	1	1	6	2	2	2	2	2
Total clusters	6	6	48	39	4	18	19	19
**Total households screened**	751	1005	1835	4614	191	2883	1267	*
Number of households with a febrile child under 5 years	272	243	1016	730	157	913	849	512
Number of households with multiple febrile cases	31	28	147	112	14	173	73	39
**Total febrile children under 5 years surveyed**	299	267	1160	852	170	1103	927	553
Number of cases receiving an antimalarial	168	124	413	309	107	523	298	166
Number of cases for whom no initial treatment was sought	13	3	34	11	5	17	91	39
Number of cases initially treated at home	161	183	436	426	165	683	231	134
Number of cases seeking care from multiple external sources	16	31	96	95	17	170	60	70
**Number of outlets screened**	1058	2336	4167	5706	566	7914	2651	4328
Number of ineligible outlets	305	1191	2348	4635	483	6455	2322	3712
Number of outlets surveyed	753	1145	1809	1068	83	1423	329	607
Number of outlets stocking ACT	177	461	1032	671	35	1287	162	301
Number of outlets stocking nAT	586	942	1687	981	82	1348	301	528
Number of outlets stocking oral AMT	41	4	958	406	26	1	23	8
								
**Number of antimalarial tablets inventoried**								
Number of ACTs	1478	3645	4378	2445	51	4908	455	969
Number of nATs	1341	1936	5668	3016	223	2545	525	808
Number of oral AMTs	54	4	1479	532	34	1	15	8

**Table B:** Characteristics of the rural household and treatment source samples by survey round and country.

	**Benin**		**Nigeria**		**Uganda**		**Zambia**	
**Survey round**	1	2	1	2	1	2	1	2
**Number of strata**	1	1	6	2	2	2	2	2
Total clusters	13	13	82	37	34	26	19	19
**Total households screened**	1561	2270	2781	3249	1872	2234	953	*
Number of households with a febrile child under 5 years	602	661	1786	580	1336	913	844	693
Number of households with multiple febrile cases	32	89	322	104	252	230	115	93
**Total febrile children under 5 years surveyed**	628	748	2114	699	1583	1171	959	788
Number of cases receiving an antimalarial	210	409	647	256	792	654	411	389
Number of cases for whom no initial treatment was sought	148	28	95	15	73	44	81	70
Number of cases initially treated at home	248	373	610	306	563	693	224	183
Number of cases seeking care from multiple external sources	20	71	206	67	126	139	59	118
**Number of outlets screened**	618	454	1289	2233	4701	8293	727	1108
Number of ineligible outlets	307	184	898	1737	3506	6467	595	853
Number of outlets surveyed	311	270	387	494	1195	1804	132	254
Number of outlets stocking ACT	124	137	136	276	580	1410	78	173
Number of outlets stocking nAT	245	199	355	452	1077	1572	123	214
Number of outlets stocking oral AMT	6	1	127	145	141	0	3	0
								
**Number of antimalarial tablets inventoried**								
Number of ACTs	574	408	236	809	1111	3862	253	477
Number of nATs	409	331	897	1273	2052	2538	202	356
Number of oral AMTs	6	1	144	165	203	0	5	0

**Table C:** Median price and IQR (2010 USD) of private sector antimalarial tablets within 5 km of households by antimalarial type and over time.

		**Urban**				**Rural**			
		**Median**	**IQR**	**p-value**	**N**	**Median**	**IQR**	**p-value**	**N**
		**Benin**							
ACT tablets	Baseline	8.14	(6.26–8.32)	0.077	267	8.44	(8.14–9.24)	<0.001	168
	Endline	7.53	(3.14–7.58)		191	2.55	(1.77–7.55)		335
									
AMT tablets	Baseline	9.83	(8.57–10.73)	0.010	121	8.29	(8.29–16.52)	0.297	64
	Endline	7.26	(7.26–7.26)		16	5.39	(5.39–5.39)		55
									
nAT tablets	Baseline	0.80	(0.39–1.03)	0.217	267	0.45	(0.26–1.45)	0.153	408
	Endline	0.90	(0.48–2.48)		201	0.53	(0.48–1.91)		415
		**Nigeria**							
									
ACT tablets	Baseline	4.94	(3.50–5.27)	<0.001	892	3.01	(2.64–3.77)	<0.001	737
	Endline	2.16	(1.35–2.32)		702	1.71	(1.35–2.16)		415
									
AMT tablets	Baseline	3.61	(3.01–3.61)	<0.001	899	3.31	(2.71–3.77)	0.003	756
	Endline	2.43	(2.16–2.59)		689	2.59	(2.37–2.80)		401
									
nAT tablets	Baseline	0.53	(0.38–0.75)	0.006	922	0.45	(0.30–0.64)	0.690	1214
	Endline	0.38	(0.32–0.54)		702	0.46	(0.40–0.54)		473
		**Uganda**							
									
ACT tablets	Baseline	5.85	(3.86–6.42)	0.055	148	3.86	(0.39–5.42)	0.266	402
	Endline	3.10	(2.32–3.87)		907	1.86	(1.55–2.32)		867
									
AMT tablets	Baseline	8.48	(7.71–9.26)	<0.001	148	9.26	(7.71–9.95)		267
	Endline	19.35	(19.35–19.35)		62	n/a	n/a		0
									
nAT tablets	Baseline	0.72	(0.47–0.72)	<0.001	148	0.47	(0.43–0.60)	<0.001	1074
	Endline	1.63	(1.08–2.44)		907	1.99	(0.77–3.25)		872
		**Zambia**							
									
ACT tablets	Baseline	8.33	(7.78–9.37)	0.001	636	7.29	(2.60–8.75)	0.385	109
	Endline	6.75	(3.91–7.83)		386	3.91	(0.00–5.87)		91
									
AMT tablets	Baseline	5.42	(5.42–6.67)	<0.001	222	n/a	n/a		0
	Endline	4.89	(4.70–4.89)		109	n/a	n/a		0
									
nAT tablets	Baseline	0.52	(0.46–0.62)	0.011	795	0.50	(0.40–0.62)	0.616	264
	Endline	0.49	(0.39–0.49)		440	0.49	(0.39–0.59)		243

This analysis is restricted to the price of antimalarial tablets as the most common dosage form used to treat uncomplicated malaria. At baseline, the median price of ACT faced by households was similar across urban and rural areas in Benin, but tended to be higher in urban areas in Nigeria, Uganda and Zambia. In all countries, the median price of ACT faced by households at baseline was many times higher than the price of nAT both in urban and rural areas; and when compared to the price of AMT, ACT tended to be cheaper in Uganda, more expensive in urban areas of Zambia, and comparable both in urban and rural areas of Benin and Nigeria.

## Appendix

See Table A.

See Table B.

See Table C.
